# Supplementary material for: TrkB-dependent regulation of molecular signaling across septal cell types
Source: Transl Psychiatry. 2024 Jan 23;14:52. doi: 10.1038/s41398-024-02758-6 (PMC10805920; doi:10.1038/s41398-024-02758-6)
Supplement: Supplementary file 1 — Supplementary Material [file 41398_2024_2758_MOESM1_ESM.docx]

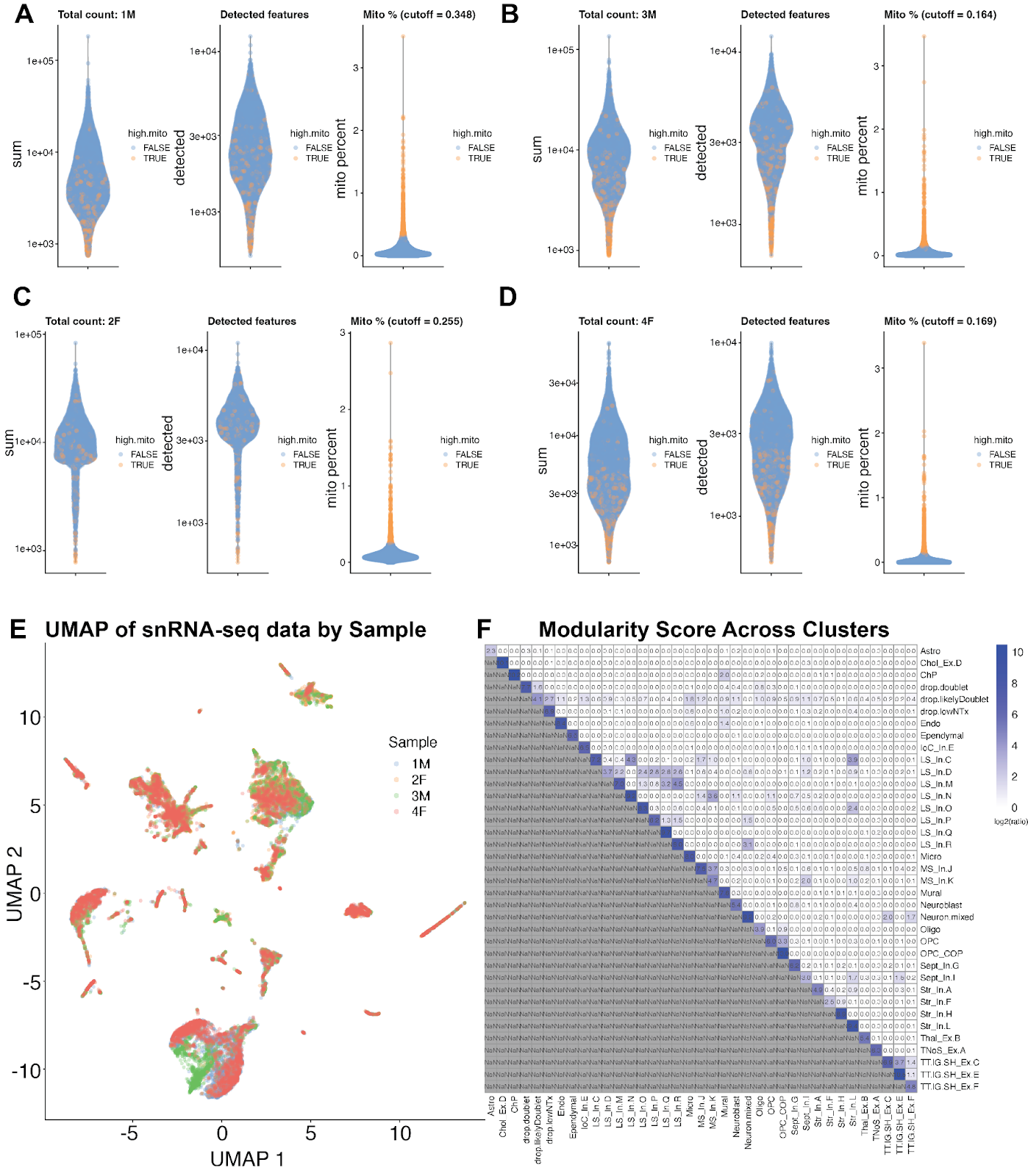


**Supplementary Figure 1: Quality control data for LS snRNA-seq samples and clusters.** Total UMI counts, genes detected, and percent of total UMIs mapping to the mitochondrial genome were plotted for the male (1M and 3M)**(A-B)** and female (2F and 4F) samples**(C-D).** Nuclei with high mitochondrial reads were removed from the data and are highlighted across all plots. **(E)** Uniform manifold approximation and projection (UMAP) of identified cell types were colored according to sample. **(F)** Modularity scores within and between clusters were calculated to assess cluster heterogeneity.**
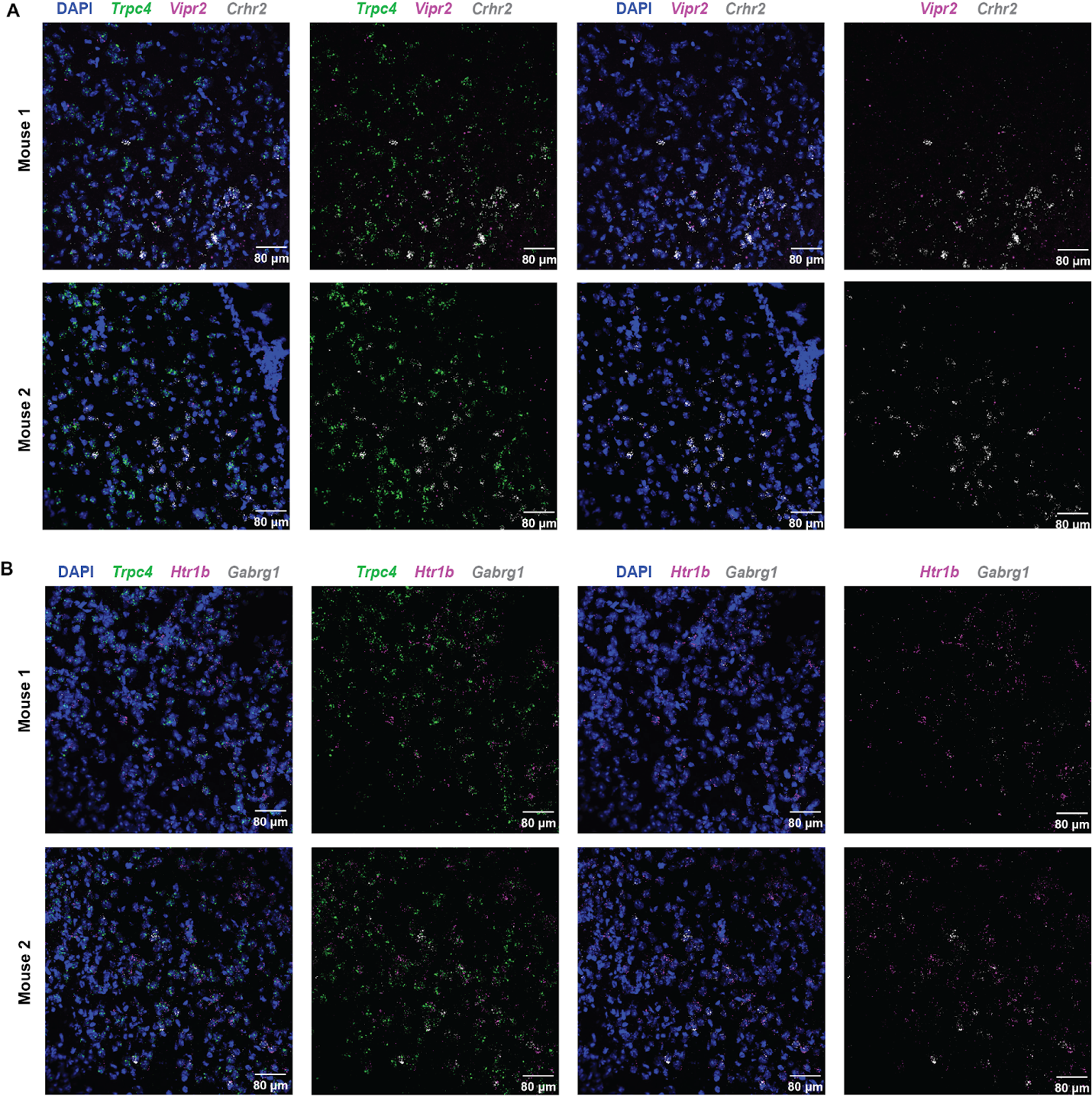
Supplementary Figure 2:** **Molecular validation of LS clusters identified with snRNA-seq.** Single molecule fluorescence *in situ* hybridization with RNAScope was performed on samples from 2 independent, adult male mice. One probe set **(A)** included the broad LS marker *Trpc4,* which overlaps with LS_In.Q marker *Crhr2* and Sept_In.G *Vipr2*. The second probe set **(B)** included the broad LS marker *Trpc4,* which overlaps with LS_In.O marker *Gabrg1* and LS_In.R marker *Htr1b*.


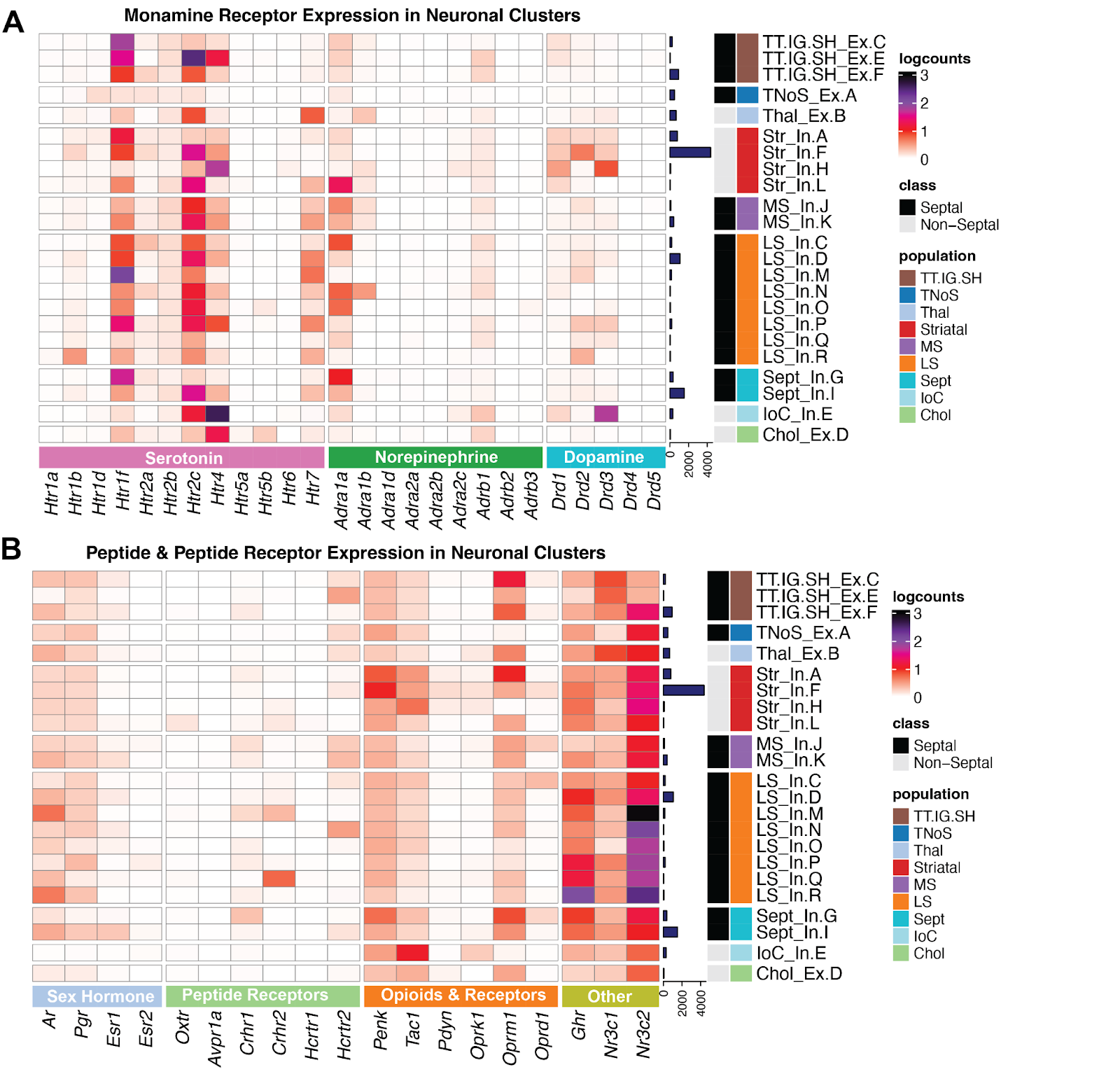


**Supplementary Figure 3: Septal clusters express a wide variety of monoamines and neuropeptides as well as neuropeptide and monoamine receptors.** Heatmaps of the (**A**) monoamine receptors and various peptides and (**B**) peptide receptors across neuronal clusters, with normalized expression values (logcounts).


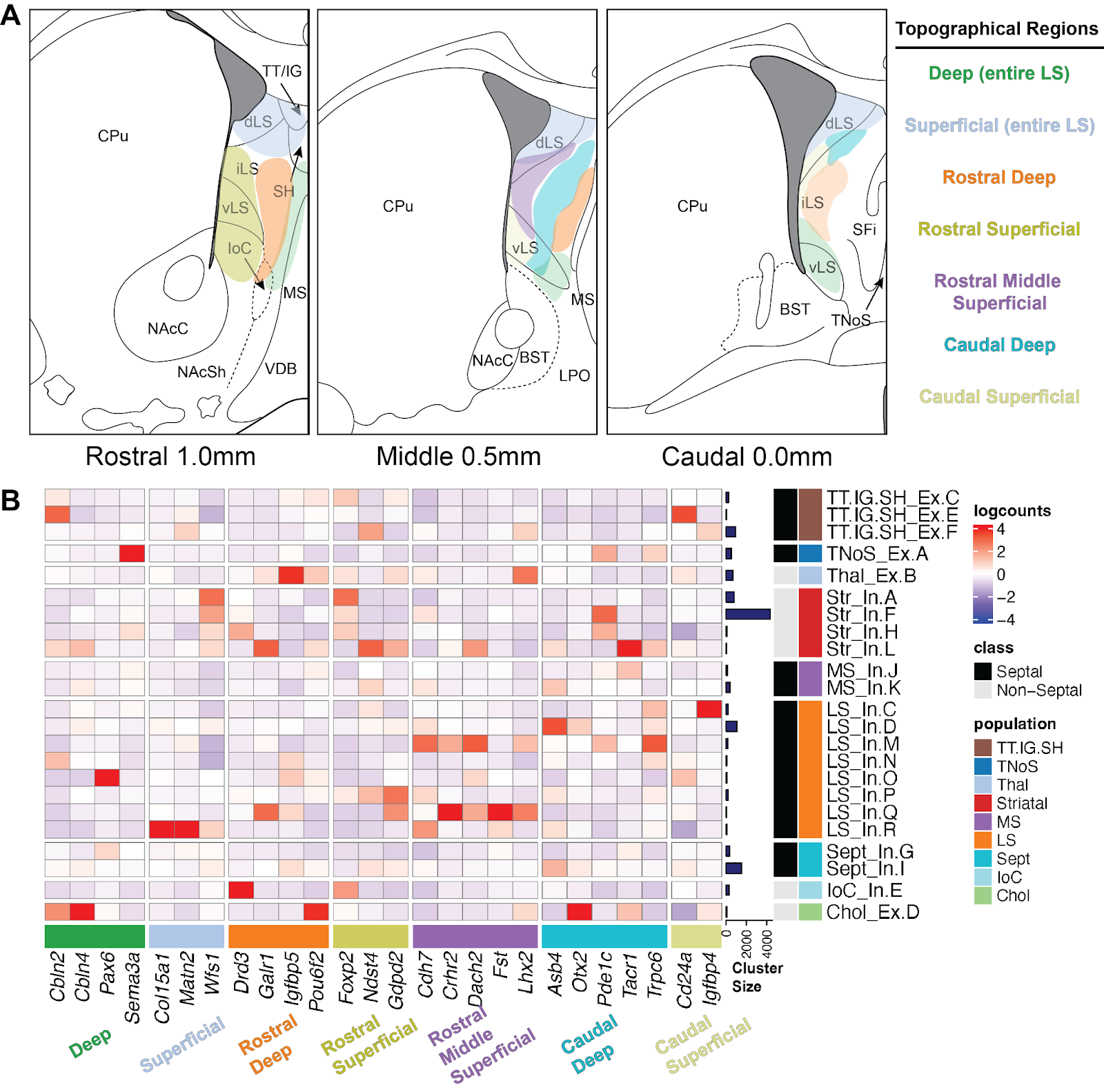


**Supplementary Figure 4: Septal neuronal clusters map onto established LS topographical markers.** (**A**) Illustration depicting the anatomical boundaries across the rostral-caudal axis of the LS, denoting the dorsal (d), intermediate (i), and ventral (v) subregions of the LS, as well as the caudate/putamen (CPu), nucleus accumbens core (NAcC) and shell (NAcSh), islands of calleja (IoC), tenia tecta/indusium griseum (TT/IG), septohippocampal nucleus (SH), medial septum (MS), bed nucleus of the stria terminalis (BST), lateral preoptic area (LPO), triangular nucleus of the septum (TNoS), and septofibrial nucleus (SFi). The illustration is color coded according to the topographical map with distinct genetic markers [^50^](https://sciwheel.com/work/citation?ids=13026226&pre=&suf=&sa=0). (**B**) Heatmap of the genetic markers for the various topographical domains in the LS across the neuronal clusters in the snRNA-seq dataset, with normalized expression values (logcounts) centered and scaled.

**
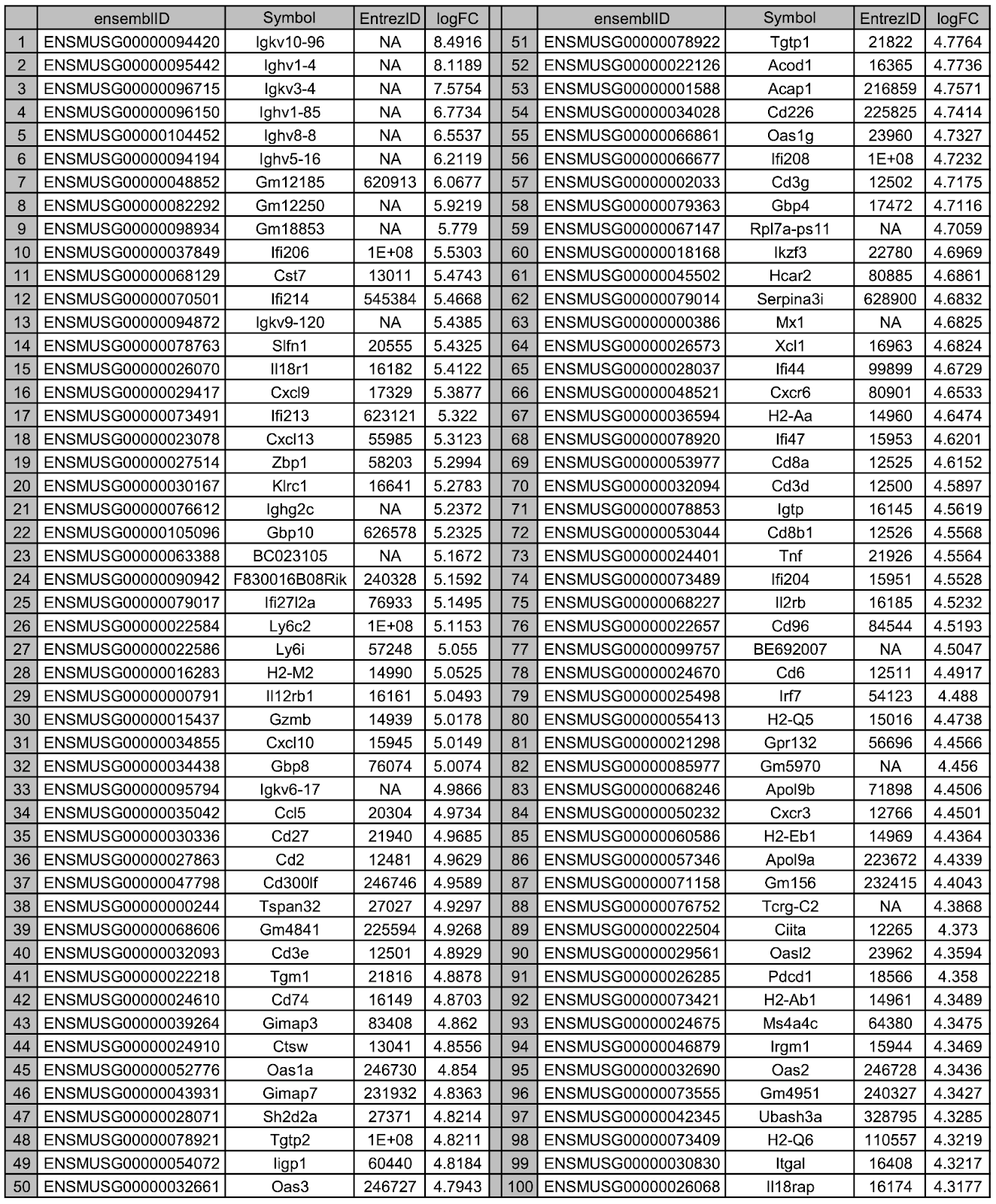
**

**Supplementary Table 1. A list of the top 100 upregulated DEGs induced by TrkB knockdown in the LS.**

**
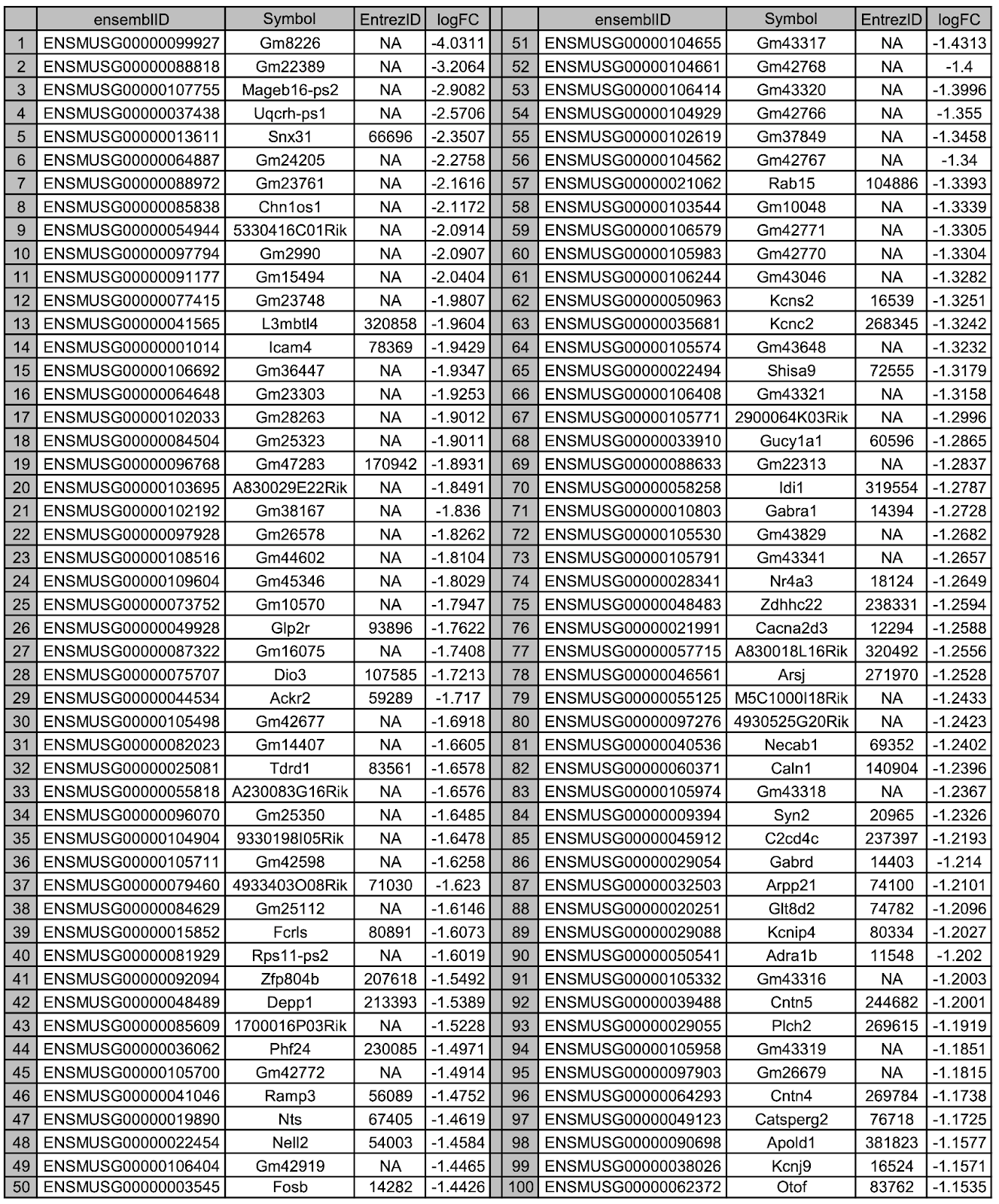
**

**Supplementary Table 2. A list of the top 100 downregulated DEGs induced by TrkB knockdown in the LS.**

**
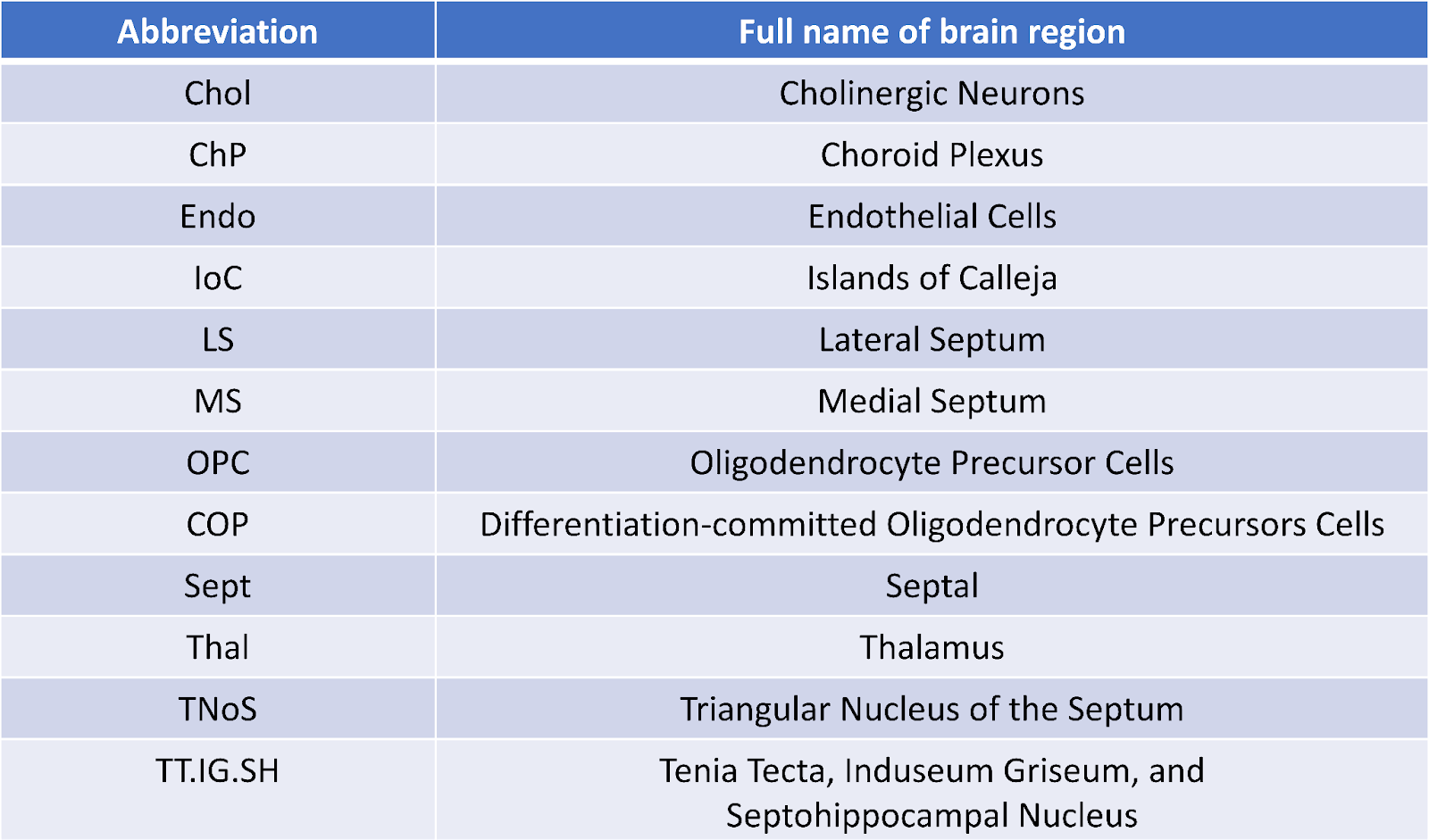


Supplementary Table 3. Abbreviations for the single-nucleus RNA-sequencing clusters.**

**
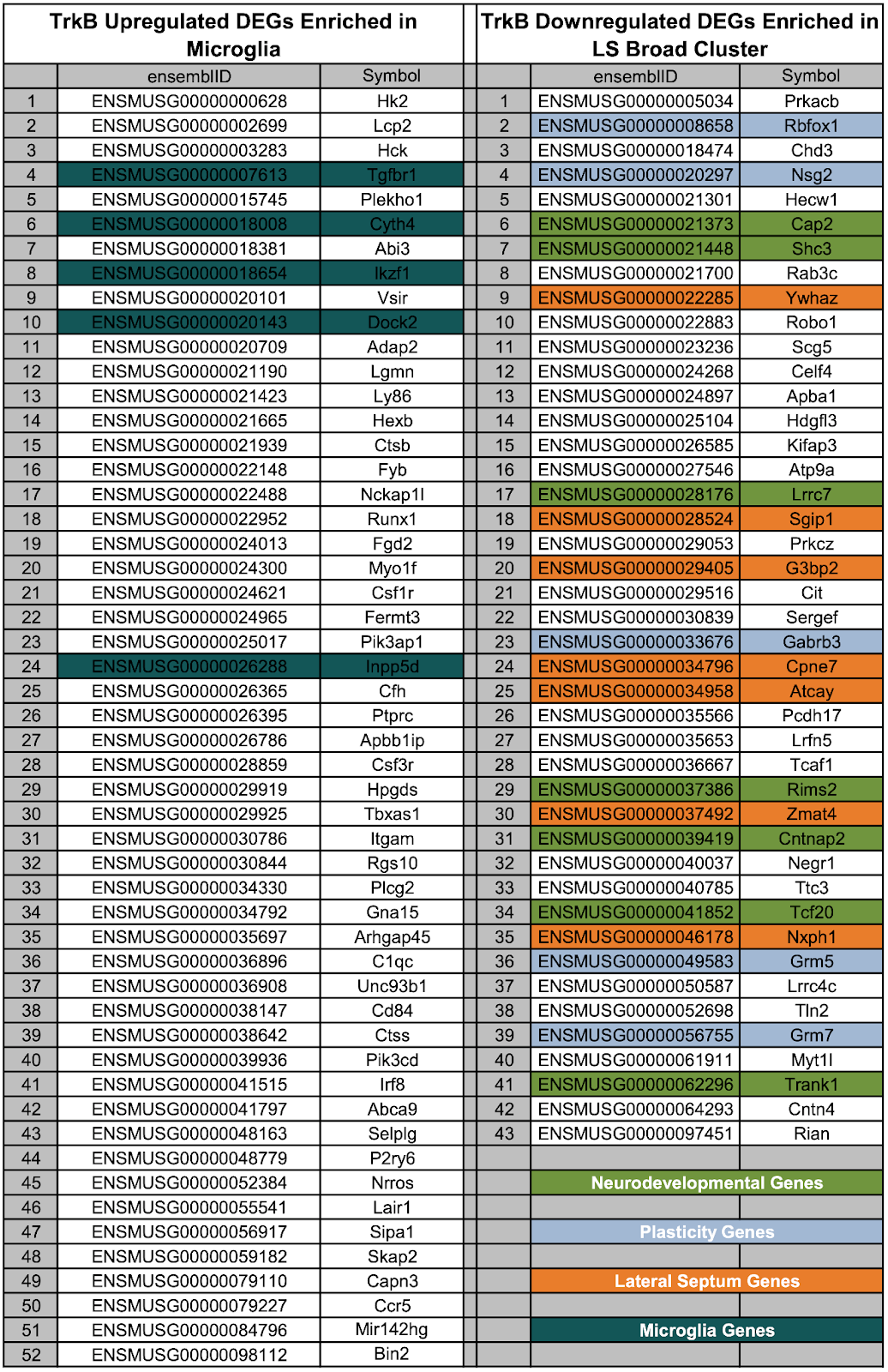
**

**Supplementary Table 4. A list of the enriched DEGs from the TrkB Knockdown dataset enriched in the microglia cluster and the broad LS cluster.**
